# Supplementary material for: Sequence context and methylation interact to shape germline mutation rate variation at CpG sites
Source: bioRxiv. 2026 May 10:2025.11.13.688199. Preprint. [Version 3] doi: 10.1101/2025.11.13.688199 (PMC13174667; doi:10.1101/2025.11.13.688199)
Supplement: Supplement 1 [file media-1.pdf]

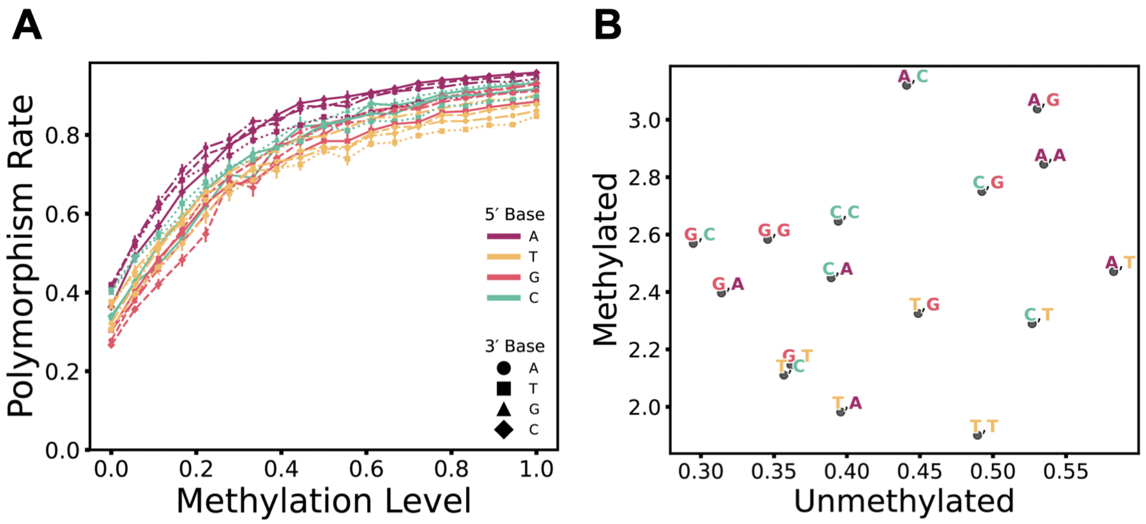

S1 Fig. Interaction between the methyl group at the focal cytosine and flanking bases on CpG mutation rate. A. Polymorphism rate for each 4-mer context, stratified by methylation levels. CpG sites were partitioned into 20 equal-width bins (ranging from [0, 0.05) to [0.95, 1]). Each point represents the proportion of SNPs among all CpG sites within a methylation bin, along with 95% confidence intervals assuming binomial sampling. Each context is represented by a color denoting the nucleotide at the 5' position and a shape denoting the nucleotide at the 3' position. **B.** Scaled mutation rates for each 4-mer context as estimated by the model for unmethylated and methylated sites. Points are labeled by the nucleotides upstream and downstream of the CpG, with error bars representing 95% confidence intervals for the estimates.

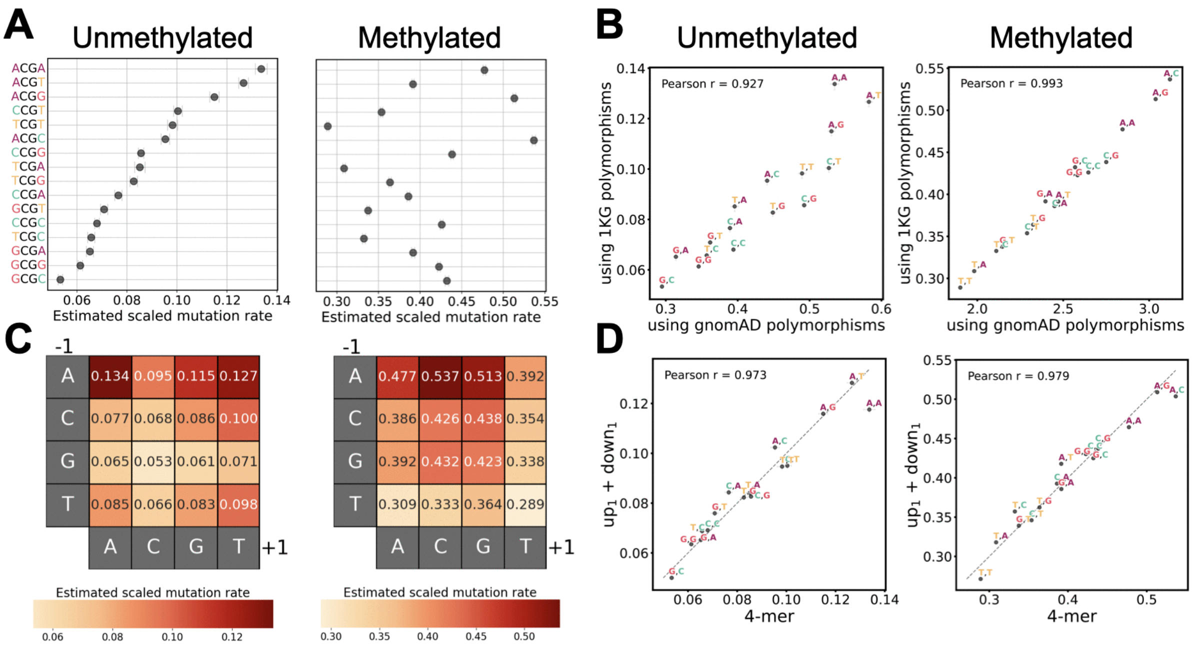

S2 Fig. Concordance between mutation rates for 4-mer CpG contexts estimated from gnomAD (v4) and 1000 Genomes (1KG) polymorphism datasets. A. Scaled mutation rate estimates for unmethylated and methylated sites for each 4-mer context using 1KG data. The contexts are sorted in descending order of mutability when unmethylated and error bars represent 95% confidence intervals for the estimates. **B.** Comparison of mutation rates estimated using polymorphisms from gnomAD and 1KG at unmethylated and methylated sites, respectively. **C.** Heatmap of scaled mutation rate estimates using the 4-mer model at unmethylated and methylated sites, using polymorphism data from 1KG. Each heatmap shows relative mutation rates for that panel; color scales are not comparable between panels. **D.** Concordance between mutation rates estimated from the 4-mer model and from the up_1+$down_1$ model using 1KG data. Points are labeled by the nucleotides upstream and downstream of the CpG, with error bars representing 95% confidence intervals for the estimates in panels B and D. The dashed line indicates $x = y$.

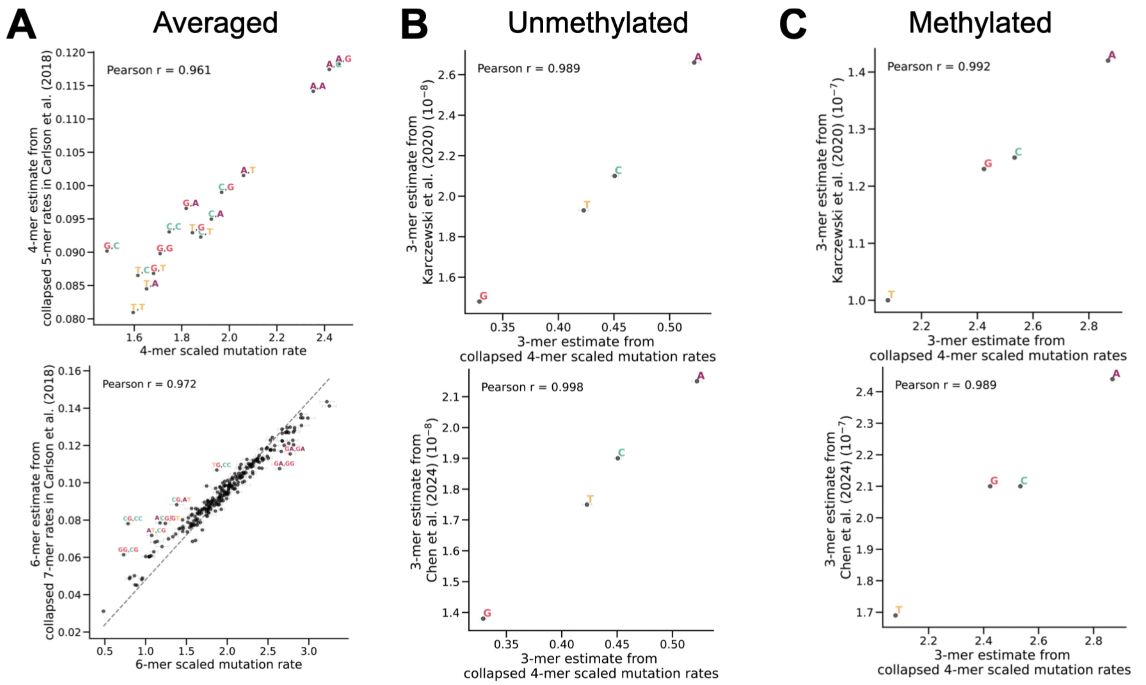

S3 Fig. Context-specific scaled mutation rates are consistent with estimates from other published context-dependent mutation models. A. Comparison with rates published in Carlson et al. (2018). To compare Carlson et al.'s estimates for 5-mers and 7-mers with our 4-mer and 6-mer results, we derived 4-mer and 6-mer rates as weighted averages of the mutation rate estimates for 5-mer (top) and 7-mer (bottom) contexts reported by Carlson et al., using the average methylation level of each 5-mer or 7-mer context as weights. For the expanded context comparison, a linear fit constrained through the origin is shown (dashed line). Only contexts with residuals exceeding 2.5 standard deviations from the fitted line are labeled. **B, C.** Comparison with rates published from gnomAD mutation rate models (Karczewski et al., 2020; Chen at al., 2024) for unmethylated (B) and methylated CpGs (C). To facilitate comparison, we computed the mutation rate for each 3-mer as the mean of estimates of the four corresponding 4-mer contexts sharing the same upstream base (e.g., rate for ACG is the mean of the rates of ACGA, ACGC, ACGG and ACGT). In all panels, points are labeled by the nucleotides upstream and downstream of the CpG, with horizontal error bars representing 95% confidence intervals for the model estimates.

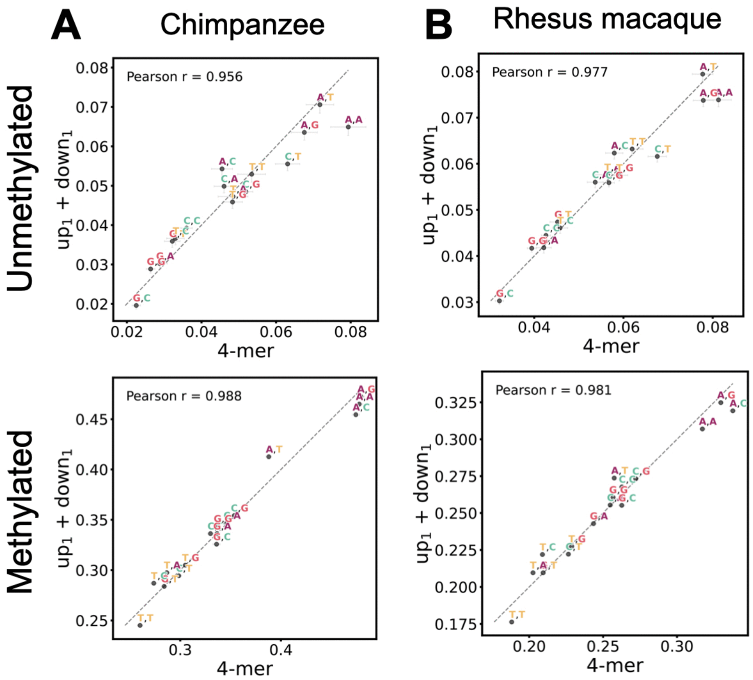

S4 Fig. Independent effects of upstream and downstream bases on CpG mutability in chimpanzee and rhesus macaque. A. Concordance between mutation rates as estimated from 4-mer model and from $up_1 + down_1$ model which assumes independent effects of upstream and downstream bases in chimpanzee for unmethylated and methylated sites. **B.** Same as (A), for rhesus macaque. Points are labeled by the upstream and downstream nucleotide flanking the CpG, and error bars denote 95% confidence intervals. The dashed line indicates $x = y$.

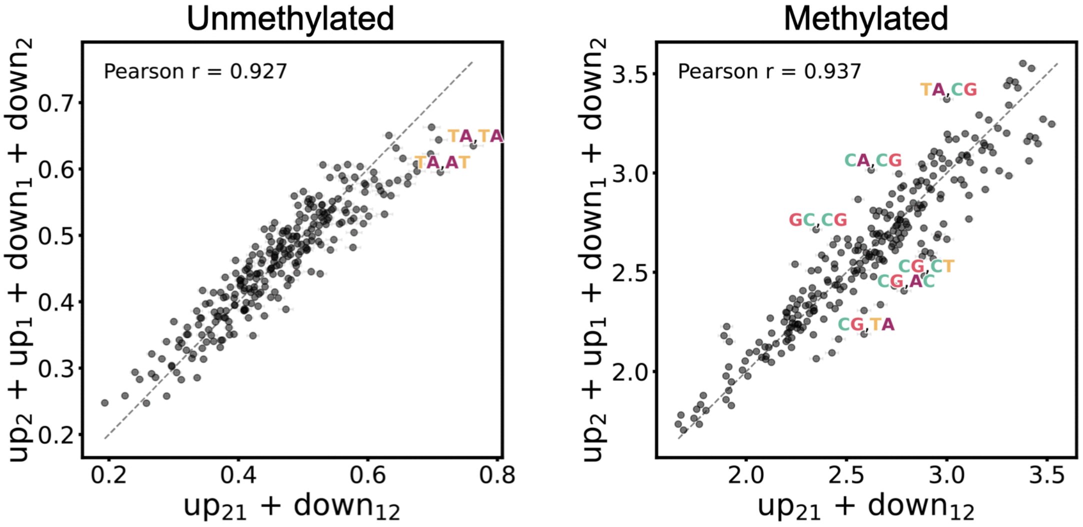

S5 Fig. Concordance in mutation rate estimates between models with and without interactions within upstream and downstream dimers. Comparison of human mutation rates as estimated from the up$_{21}$+down$_{12}$ model, which assumes interactions between bases within the dimer upstream and downstream to the CpG, and the up$_2$+up$_1$+down$_1$+down$_2$ model, which assumes independent effects of every flanking base. Only contexts for which the difference between the two models' estimates is more than 2.5 standard deviations away from the average difference across all contexts are labelled. Error bars represent 95% confidence intervals for the estimates on both axes. The dashed line indicates $x = y$.

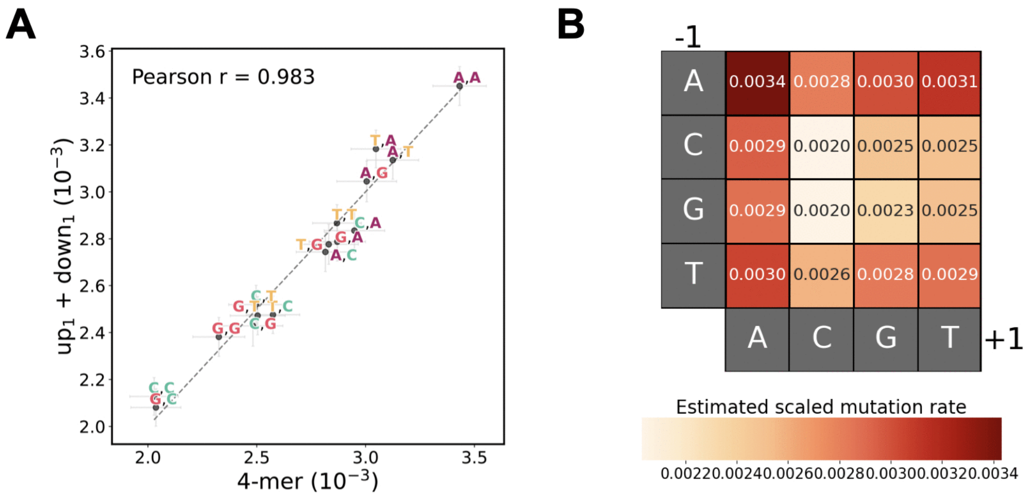

S6 Fig. Independent effects of upstream and downstream bases on CpG mutability in the insect species, *Bombyx mori* (silkworm). A. Concordance between mutation rates as estimated from 4-mer model and from up_1+$down_1$ model. Mutation rate is modelled as a function of sequence context alone**.** Points are labeled by the upstream and downstream nucleotide flanking the CpG, and error bars denote 95% confidence intervals. The dashed line indicates $x = y$. **B.** Heatmap of estimated scaled mutation rates using the 4-mer model.

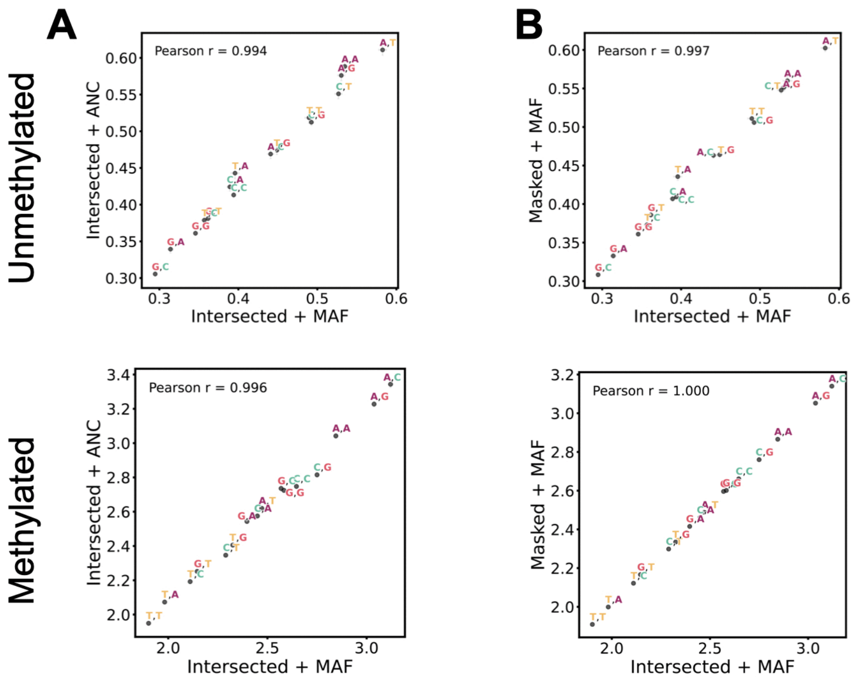

S7 Fig. Context-specific scaled mutation rate estimates are highly concordant regardless of CpG filtering or SNP polarization method. A. Estimates obtained by polarizing SNPs using minor allele frequency (MAF) are compared to those based on the inferred ancestral allele (ANC) for CpG sites present in both the hard-masked reference and inferred ancestral genome. **B.** Estimates derived from the intersected reference genome—restricted to CpGs present in both the hard-masked reference and inferred ancestral genome—are compared to those from the masked reference genome without intersection ("Masked"); SNPs are polarized by MAF. Points are labeled by the upstream and downstream nucleotide context flanking the CpG, and error bars denote 95% confidence intervals around the estimates.

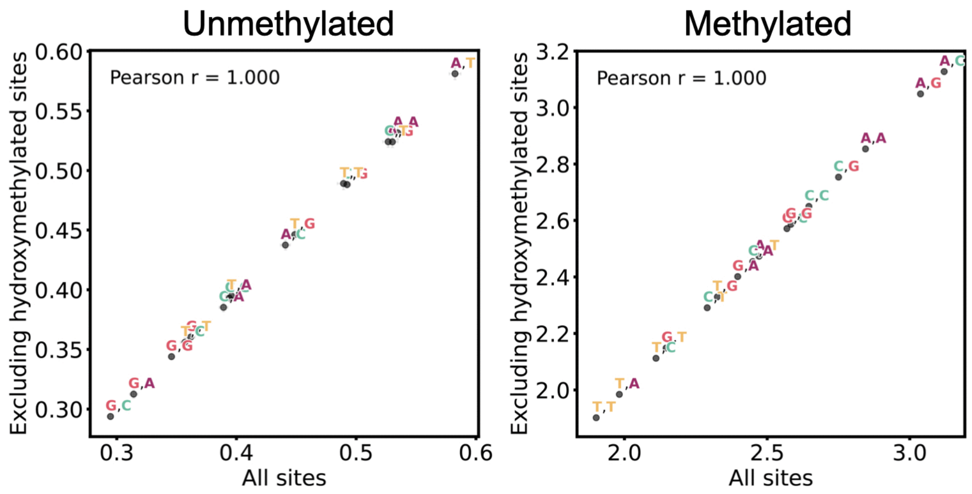

S8 Fig. **Scaled mutation rate estimates are nearly perfectly concordant after excluding hydroxy-methylated (5hmC) sites.** Comparison of the model's scaled mutation rate estimates for unmethylated and methylated sites when using all sites and when excluding sites with a non-zero 5hmC level as measured in human ES-cells. Points are labeled by the upstream and downstream nucleotide context flanking the CpG, and error bars denote 95% confidence intervals around the estimates.

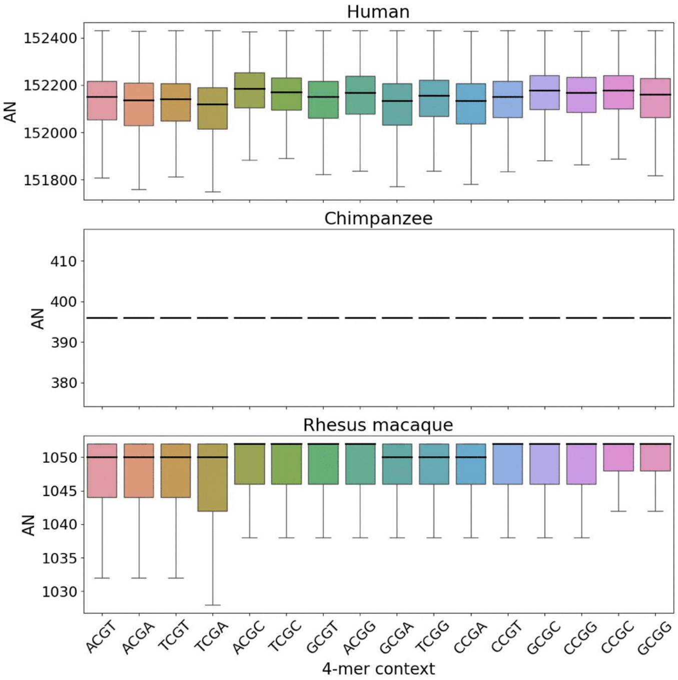

S9 Fig. Callable allele coverage across 4-mer contexts in primate polymorphism datasets. Distribution of the number of callable alleles by 4-mer sequence context in the human, chimpanzee, and rhesus macaque polymorphism dataset. Boxes show the median and interquartile range, with whiskers extending to 1.5x the interquartile range.

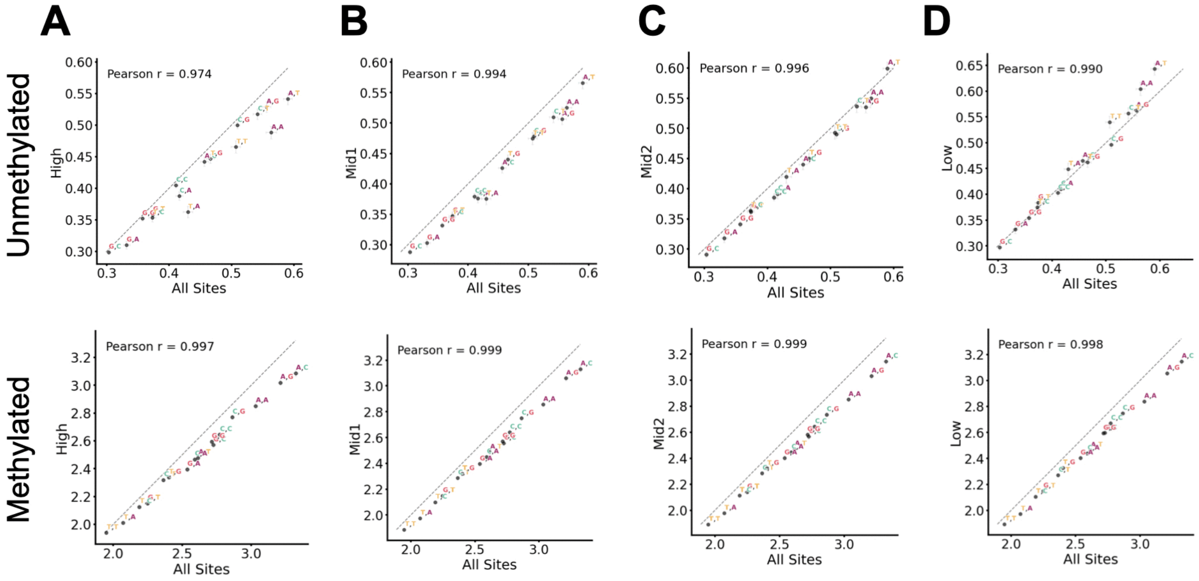

S10 Fig. Context-specific scaled mutation rate estimates are consistent across recombination rate strata. A. Comparison of scaled mutation rate estimates across all sites versus sites stratified by recombination rate, shown separately for unmethylated (top) and methylated (bottom) CpGs. Panels correspond to recombination rate quartiles: **A.** highest (“High”, > 0.389), **B** upper-middle (“Mid1”, ≤ 0.389), **C.** lower-middle (“Mid2”, ≤ 0.026), and **D.** lowest (“Low”, ≤ 4.877 × 10^{-5}). The dashed line indicates $x = y$. Points are labeled by the upstream and downstream nucleotide flanking the CpG, and error bars denote 95% confidence intervals.

S1 Table. Scaled mutation rate estimates for each 4-mer and 6-mer CpG context at unmethylated and methylated sites (provided as excel file). This table contains separate worksheets for each species and model (e.g., 4-mer, 6-mer), with each sheet reporting the sequence context, methylation status, dataset-specific scaled mutation rate estimate (μT), and its associated standard error. For silkworm, we report estimates from 4-mer and up_1+$down_1$ models only, and rates are modeled as a function of context alone.

Species	Model	Null Deviance	Residual Deviance	Parameters	AIC	BIC	Proportion Variance Explained (%)
Human	4-mer	6,405,823	4,793,103	32	4,793,167	4,793,604	25.18%
	up$_1$+down$_1$	6,405,823	4,795,460	14	4,795,488	4,795,679	25.14%
	6-mer	6,405,823	4,771,028	512	4,772,052	4,779,036	25.52%
	up$_{21}$+down$_{12}$	6,405,823	4,776,600	62	4,776,724	4,777,569	25.43%
	up$_2$+up$_1$+down$_1$+down$_2$	6,405,823	4,783,251	26	4,783,303	4,783,658	25.33%
Chimpanzee	4-mer	4,464,404	4,235,923	32	4,235,987	4,236,410	5.12%
	up$_1$+down$_1$	4,464,404	4,237,096	14	4,237,124	4,237,309	5.09%
	6-mer	4,464,404	4,224,537	512	4,225,561	4,232,342	5.37%
	up$_{21}$+down$_{12}$	4,464,404	4,227,288	62	4,227,412	4,228,233	5.31%
	up$_2$+up$_1$+down$_1$+down$_2$	4,464,404	4,230,348	26	4,230,400	4,230,744	5.24%
Rhesus macaque	4-mer	8,809,643	8,516,789	32	8,516,853	8,517,303	3.32%
	up$_1$+down$_1$	8,809,643	8,518,893	14	8,518,921	8,519,118	3.30%
	6-mer	8,809,643	8,498,697	512	8,499,721	8,506,911	3.53%
	up$_{21}$+down$_{12}$	8,809,643	8,503,413	62	8,503,537	8,504,407	3.48%
	up$_2$+up$_1$+down$_1$+down$_2$	8,809,643	8,509,065	26	8,509,117	8,509,482	3.41%
Silkworm	4-mer	408,193	407,611	16	407,643	407,870	0.143%
	up$_1$+down$_1$	408,193	407,630	7	407,644	407,743	0.138%

S2 Table. Model comparison for human and non-human species. Summary statistics of each fitted regression model for the four species in our analysis. The proportion variance explained is calculated as the difference between null and residual deviance divided by the null deviance. In silkworm, the mutation rate is modeled as a function of sequence context alone.

**Human**	
Number of individuals	807,162
Number of non-genic, non-conserved CpG sites	6,710,813
Number of non-genic, non-conserved CpG C>T SNPs	4,953,613
**Chimpanzee**	
Number of individuals	198
Number of non-genic CpG sites	8,523,730
Number of non-genic CpG C>T SNPs	958,654
**Rhesus macaque**	
Number of individuals	526
Number of non-genic CpG sites	9,505,961
Number of non-genic CpG C>T SNPs	1,714,326
**Silkworm**	
Number of individuals	1,082
Number of non-genic CpG sites	10,767,630
Number of non-genic CpG C>T SNPs	29,600

S3 Table. Summary of CpG sites and SNPs for each species. Genic regions are excluded in our analysis using NCBI *RefSeq* annotations. For human, phylogenetically conserved regions as defined by *phastcons* are also excluded. We identify C>T SNPs by polarizing the substitution that gave rise to each SNP using minor allele frequencies for human, chimpanzee, and rhesus macaque. For silkworm, we used *est-sfs*, a method that infers the ancestral state probabilities at polymorphic sites using information from a focal and outgroup species.

Dataset	Null Deviance	Residual Deviance	Proportion Variance Explained (%)	Number of neutral sites	Number of CpG>TpG SNPs
Human (Intersected, MAF)	6,405,823	4,793,103	25.18%	6,710,813	4,953,613
Human (Masked, MAF)	7,418,266	5,635,875	24.03%	8,176,931	5,908,808
Human (Intersected, ANC)	6,389,305	4,798,654	24.90%	6,710,813	4,959,972
Chimpanzee (Intersected, MAF)	4,464,404	4,235,923	5.12%	8,523,730	958,654
Chimpanzee (Masked, MAF)	4,866,736	4,616,709	5.14%	10,010,745	1,046,806
Chimpanzee (Intersected, ANC)	4,294,376	4,083,915	4.90%	8,523,730	891,262
Rhesus macaque (Intersected, MAF)	8,809,643	8,516,789	3.32%	9,505,961	1,714,326
Rhesus macaque (Masked, MAF)	9,579,179	9,248,147	3.46%	10,432,387	1,860,329
Rhesus macaque (Intersected, ANC)	6,925,334	6,761,980	2.36%	9,505,961	1,158,913

S4 Table. Model comparison across CpG filtering and SNP polarization methods in human and non-human species. Summary statistics of the 4-mer fitted regression model for each of the three primates in our analysis using the following combinations of CpG filtering and SNP polarization methods: intersected reference genome (CpGs present in both the hard-masked reference and inferred ancestral genome) and minor allele frequency (MAF) polarization; hard-masked reference genome and MAF polarization; intersected reference genome and ancestral allele polarization (ANC). The proportion variance explained is calculated as the difference between null and residual deviance, divided by the null deviance. We also show the number of CpG sites extracted from each genomic build after excluding genic regions and conserved regions (for human) and the number of CpG>TpG SNPs inferred using the corresponding polarization method.
